# Are you having a LaPh? Diverse roles of Labile Phosphorylation in mammalian cells

**DOI:** 10.1042/BST20253100

**Published:** 2025-10-22

**Authors:** Christopher J. Clarke, Claire E. Eyers

**Affiliations:** Centre for Proteome Research, Institute of Systems, Molecular and Integrative Biology, University of Liverpool, Crown Street, Liverpool, L69 7ZB, U.K.

**Keywords:** atypical, labile, non-canonical, phosphorylation

## Abstract

Phosphorylation plays a central role in regulating signal transduction across all kingdoms of life, allowing organisms to sense and respond to their environment. In mammals, the signalling research field is dominated by the functions of pSer, pThr and pTyr, due to both historical and technological factors. Mostly ignored are the labile phosphosites (LaPhs), made up of six other phosphorylatable amino acids: His, Lys, Arg, Asp, Glu and Cys. This group is characterised by an acid and/or heat-labile phosphate linkage, forming a distinct group from the highly stable phosphomonoesters of pSer, pThr and pTyr. LaPhs have distinct thermal and pH stability profiles, which may contribute to, or even dictate, their functions. Here, we review the contribution of LaPhs to mammalian signalling networks, highlighting their currently defined diverse functions.

## Introduction

**Figure 1:**
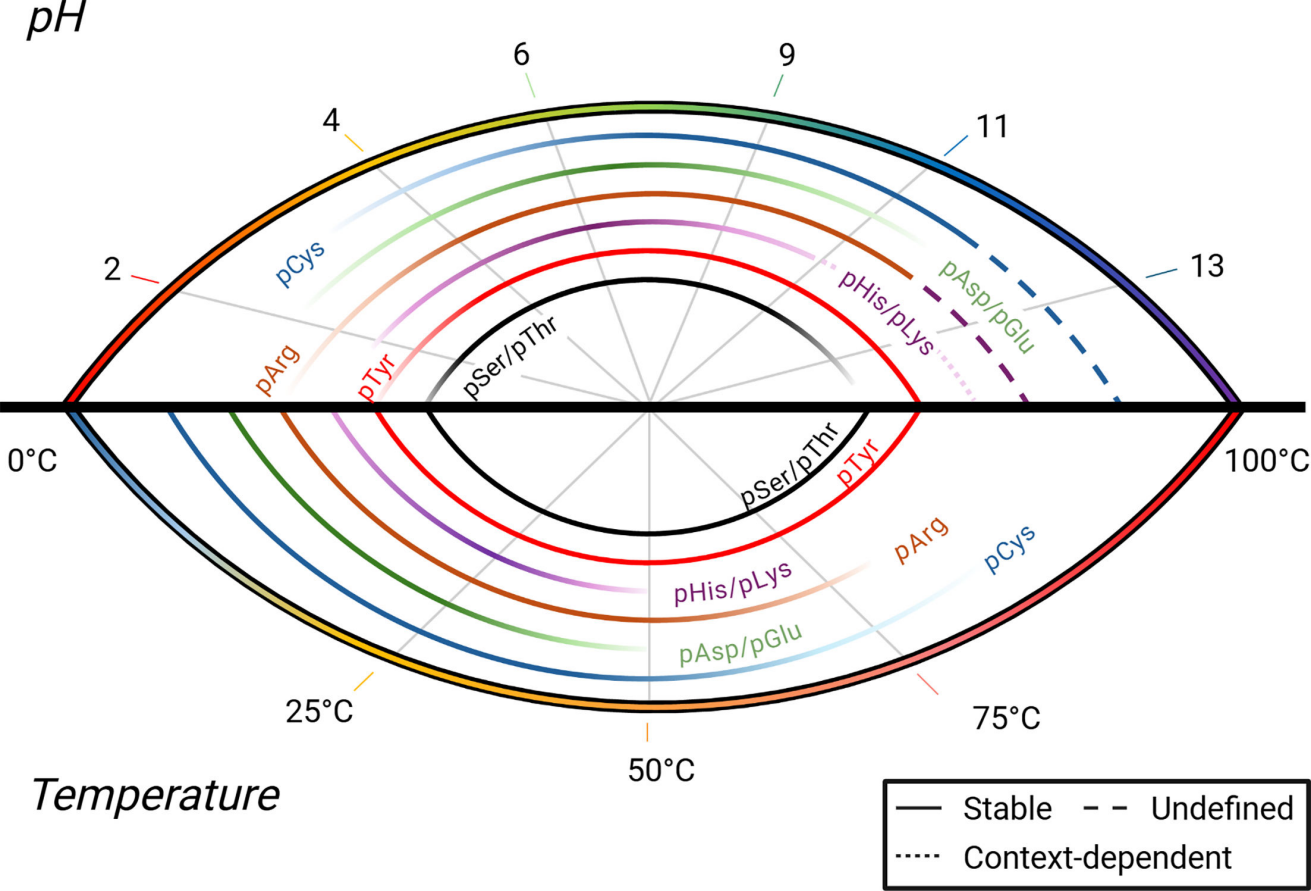
Stability landscape of labile and non-labile phosphoresidues. Experimentally derived stabilities for phosphoresidues as a function of pH (1–14, top panel) and temperature (0-100°C, lower panel) are shown. No experimental evidence for the stability of pArg or pCys could be found for pH exceeding 12. Stabilities are reported from the following studies: pSer/pThr* [2], pTyr^£^ [3,4], pHis/pLys^#^ [5–7], pArg [8,9], pAsp/pGlu^£^ [10–12], pCys^#^ [13–15]. Values derived from: *peptide-incorporated phospho-amino acid, ^£^free phospho-amino acid and peptide-incorporated phospho-amino acid, ^#^peptide- and protein-incorporated phospho-amino acid. For pHis, studies demonstrate different stabilities at higher pH, suggesting stability may be context-dependent. Created in https://BioRender.com.

The reversible, covalent attachment of phosphate to amino acid side chains in proteins is a staple of mammalian signalling systems, with an estimated 82,000 confident sites of phosphorylation in the human proteome described to date [1]. This number encompasses only phosphorylation on Ser, Thr and Tyr residues (so-called typical or canonical phosphorylation), which are characterised by a highly stable phosphomonoester linkage. However, six other residues, His, Lys, Arg, Asp, Glu and Cys, display evidence of phosphorylation across different organisms. These sites are characterised by varying degrees of heat- and pH-lability (Figure 1) and make up what we term labile phosphorylation sites (LaPhs), also known as the atypical or non-canonical class of phospho-amino acids. While other phosphate-based post-translational modifications (PTMs) have also been reported, including pyrophosphorylation of Ser [16–19], polyphosphorylation of Lys [20–23] and His [24–26], and phosphorylation of hydroxy-Pro [27,28] and hydroxy-Lys [29,30], this review focusses on the addition of a single phosphate group to the non-canonical standard amino acids.

The characteristic lability of LaPhs has made their investigation challenging, particularly in the context of high-throughput mass spectrometry (MS) experiments. We have recently reviewed these challenges in depth [31], but it is worth noting here that traditional MS-based workflows for phosphoproteomic analyses typically utilise high temperatures and extremes of pH to aid sample extraction and enhance the specificity of phosphopeptide isolation, both of which can induce LaPh hydrolysis (Figure 1). Compounding this is the lability of LaPh modifications during (tandem) MS analysis, although this can partially be overcome using alternative, ‘softer’ fragmentation methods. However, application of alternative fragmentation regimes typically comes at the expense of depth of coverage due to time constraints. Global phosphopeptide investigations can therefore be heavily biased against LaPhs, particularly in mammalian systems where evolution has favoured pSer as the predominant phospho-signalling moiety, occurring ~5–8 times more frequently than pThr or pTyr [1], and by our estimation, around 10–30 higher than any individual LaPh residue [5]. Thus, even with some LaPh events having been described on human proteins as early as the 1960s [32], large-scale investigation has been hampered by a number of factors: i) the extreme prevalence of pSer in mammalian cells masking LaPh sites, ii) the heat- and acid-lability of LaPhs, compromising detection using both traditional high throughput (i.e. MS) and low-throughput (e.g. immunoblot) experiments, and iii) the simple belief that LaPhs do not occur and/or are not relevant for signalling in mammalian systems, meaning that their presence has generally not been considered.

Interest in mammalian LaPh signalling has been rising steadily over the last decade as more studies demonstrate their prevalence and biological relevance and their potential contribution or elevation in many disease states [33–36]. Consequently, the number of tools for their investigation, particularly pHis, is also growing [37]. In this review, we showcase the current landscape of LaPh-mediated signalling in mammalian cells (Table 1), and speculate on future areas of interest for investigation.

**Table 1:** Key effectors and substrates of LaPh phosphorylation, and their associated (or proposed*) biological roles.

Phosphorylation type	Residue(s)	Biological roles	Key effectors	Key substrates
Basic residue phosphorylation	His, Lys, Arg	Energy metabolism, ion channel regulation, proliferation, RNA processing*, epigenetics/gene expression*	NME1/NME2 (Nucleoside diphosphate kinase A/B), PHPT1 (phosphohistidine phosphatase 1), PGAM5 (phosphoglycerate mutase family member 5), LHPP (phospholysine phosphohistidine inorganic pyrophosphate phosphatase)	Annexin A1, ATP-citrate lyase, focal adhesion kinase, histones (H1, H3, H4), succinic thiokinase, KCa3.1 (intermediate conductance calcium-activated potassium channel protein 4), transducin, TRPV5 (transient receptor potential cation channel subfamily V member 5)
Acidic residue phosphorylation	Asp, Glu	Ion transport, ribosome biogenesis, nuclear energy storage, RNA processing*	P-type ATPases (SERCA1a (sarcoplasmic/endoplasmic reticulum Ca-ATPase 1a)), Rio1/Rio2, NME1	Prothymosin α, aldolase C, collagen α2
Redox-sensitive phosphorylation	Cys	Mg^2+^ homeostasis, mitophagy*, hypoxic response*	PRL3 (phosphatases of regenerating liver), PINK1 (PTEN-induced kinase 1)	HIF1α (hypoxia-inducible factor 1α)

### Phosphorylation of basic residues: pHis, pLys and pArg

#### **pHis** signalling

His (and Asp) phosphorylation is a staple of prokaryotic, fungal and plant signalling (reviewed in [38]). However, the existence of significant mammalian signalling mediated by LaPhs, and pHis in particular, remains somewhat controversial. Over recent years, numerous high-throughput proteomics studies have identified extensive pHis-containing proteins or mapped pHis sites on mammalian proteins, albeit using slightly different approaches [5,39–41]. Yet, a notable proteomics investigation of digested HEK293T cell extracts by Leijten and co-workers reported only four confidently identified pHis sites (compared with 23 in *E. coli*), suggesting that pHis does not exist at a scale that would suggest substantive signalling effects [42]. Crucially, these data were subject to very stringent filtering criteria, removing over 99% of the 1050 sites initially identified, and failing to identify some well-characterised pHis sites. On the other hand, our own early work in this area estimates the propensity of pHis in HeLa cells to be similar to that of pTyr, with 129 and 138 sites of pHis and pTyr being identified with broadly comparable false localisation rates [5]. Given that we currently estimate ~12k Tyr phosphosites in the human phosphoproteome (based on computed false discovery rates within publicly available proteomics repositories [1]), it is possible that the total number of human pHis sites may exceed 5k.

His is unique among the LaPhs in that it can exist as two isoforms (1-pHis or 3-pHis), depending on which nitrogen in the imidazole ring is phosphorylated [43]. Using isoform-specific antibodies, Fuhs et al. revealed distinct protein cohorts for these two isoforms, suggesting isoform-specific functions, either as enzyme intermediates or signalling regulators, in mammalian protein regulation [40]. The different functional effects of these two pHis isoforms may in part be due to differences in their relative stability: 1-pHis has a notably shorter half-life than 3-pHis. While both catalytic phospho-intermediates and the longer lasting phosphorylation-signalling events have both been identified in MS-based proteomics investigations, it is worth noting that the timescale of analysis and the rapid on-off nature of enzyme intermediates means that capturing more stable signalling events is much more likely. As pHis datasets expand, it will be interesting to see what proportion are defined as 3- versus 1-pHis isoforms and whether they function to drive signalling outputs or act as catalytic intermediates. Interestingly, there is evidence that phosphorylation within a catalytic core can also drive signalling events [see the discussion below about phosphatase of regenerating liver (PRL) kinases], so the two should not necessarily be considered mutually exclusive.

The scale of mammalian pHis proposed above is broadly supported by an NMR study from Makwana et al. [44], which determined the absolute amount of protein-bound pHis (specifically, the 3-pHis isoform) in a human bronchial epithelial cell line (16HBE14o) to be 1.8 fmol/cell, ~3.2fold less than the measured amount of protein-bound pSer and pThr (5.8 fmol/cell combined). It should be noted that the measured 3-pHis signal was near the limit of detection in these experiments, and while the protein source and cellular distribution of pHis could not be determined, the levels observed strongly suggest that this PTM is likely to be important for mammalian biology. The apparent disparity between the relatively high proportion of cellular protein His phosphorylation detected by NMR and the relatively few known mammalian histidine kinases (see below) may reflect that mammalian His (and potentially other LaPh) phosphorylation events are not necessarily enzymatically mediated. His residues can be chemically phosphorylated *in vitro* with relative ease [45], and based on our own observations, non-enzymatic phosphate transfer to His can occur in cellular extracts (data not shown), from peptides in solution and during the analysis [46]. Whether protein pHis occurs at this scale in cells or is elevated due to phosphate transfer during sample preparation or analysis remains to be determined.

Proteomic and functional investigations to date indicate that pHis may be involved in metabolism, transcription, RNA processing and cell cycle progression, although some of these assigned functions have yet to be validated. Perhaps the most well-defined roles for pHis in mammals then revolve around the histidine phosphotransferases/kinases NME1 and NME2 [nucleotide diphosphate kinase (NDPK)-A and NDPK-B, respectively]. Both of these enzymes play roles in cellular energy homeostasis, maintaining a stable distribution of nucleoside triphosphates by transferring a phosphate group from a triphosphate (e.g. ATP) to a diphosphate (e.g. GDP), yielding a new triphosphate molecule (e.g. GTP) [36]. Transfer of this phosphate group occurs through a critical 1-pHis intermediate [47], such that mutation of this catalytic residue (i.e. His118 in NME1) eliminates its phosphotransferase activity [48]. NME1 also has several known protein substrates, including ATP-citrate lyase [49] and succinic thiokinase (succinyl-CoA synthetase) [48], which are phosphorylated on His residues that form intermediates in their own catalytic cycles. This could ostensibly link levels of cellular nucleoside di/triphosphates to energy production, marking out NME1 as an ATP sensor. Annexin A1, known for its roles in apoptosis and as an anti-inflammatory agent [50], is also a reported substrate of NME1 [51,52], where His phosphorylation is thought to promote proteolytic release of a C-terminal fragment, in turn resulting in annexin A1 degradation. This could clearly have implications for cancers and a variety of inflammatory conditions, and indeed NME1 expression has been shown to correlate with oesophageal squamous cell carcinoma progression [53], where its phosphorylation of focal adhesion kinase (FAK) on His58 is required for growth factor-independent tumour cell growth. Regulation of annexin A1 and FAK by NME1-dependent His phosphorylation thus ties pHis to a number of vital biological processes.

Interestingly, while pHis appears to be broadly distributed across the cell (based on immunofluorescence studies [40]), the small repertoire of NME2 substrates identified to date are all membrane associated, with NME2 inducing His phosphorylation of calcium channel TRPV5 (transient receptor potential cation channel subfamily V member 5) [54], potassium channel K_Ca_3.1 [55,56] and the Gβ_1_ subunit of trimeric G proteins [57]. In all three of these examples, His phosphorylation appears to mediate channel/receptor activity. In the latter, NME2-mediated phosphorylation of His266 in the β_1_ subunit [57] facilitates GDP to GTP exchange in the α subunit, amplifying the response to GPCR (G-protein couple receptor) activation. In CD4 T-cells, calmodulin associates with the K_Ca_3.1 channel through a calmodulin-binding domain [33] and under basal conditions, Cu^2+^ ions are co-ordinated through His358 in each of the four channel subunit tails, which prevents the conformational change required for K^+^ efflux [56]. In the presence of increased intracellular Ca^2+^, a conformational change in the receptor-associated calmodulin induces a conformational change in K_Ca_3.1 itself, allowing NME2 to access and phosphorylate His358. This now disrupts the His-Cu^2+^ interaction, allowing the channel to open and resulting in T-cell activation.

#### pLys and pArg signalling

In contrast with pHis, little is known about the roles of pLys and pArg in human cells, with only a handful of high-throughput studies reporting their identification [5,58,59], none of which explored the functional effect at the individual protein level. Our own work has identified pLys and pArg in proteins from human cells at a similar scale to that observed for pTyr [5], with the number of pArg sites being comparable with those reported in a study by Fu et al. [59]. While that study (and our unpublished observations) suggests putative roles for pArg in nucleic acid binding, these data currently need to be tempered given the relatively low site localisation confidence in both datasets. However, our hypothesis as to putative function is supported by previous observations of an arginine-specific protein kinase tightly bound to rat liver DNA [60], and that histone H3 can be phosphorylated *in vitro* on multiple arginine residues by a kinase partially purified from nuclear extracts of mouse leukaemia cells, in a Ca^2+^-calmodulin-dependent manner [61]. When considered in the context of observed pHis on histone H4 [62] and pLys on histone H1 [63,64], Arg phosphorylation on histone H3 hints at more generalised roles for basic residue phosphorylation in the regulation of gene expression.

#### Phosphatase-based regulation of pHis, pLys and pArg signalling

Lending more weight to the hypothesis of extensive functional roles for pHis in mammalian systems are the three histidine phosphatases that have thus far been described: PHPT1 (phosphohistidine phosphatase 1), PGAM5 (phosphoglycerate mutase family member 5) and LHPP (phospholysine phosphohistidine inorganic pyrophosphate phosphatase). PHPT1 is known to dephosphorylate pHis on ATP-citrate lyase [65], histone H4 [66], Gβ and Gγ subunits of the GPCR transducin [67], and potassium channel K_Ca_3.1 [68]. Srivastava and colleagues demonstrated that overexpression of PHPT1 inhibits activity of the K_Ca_3.1 channel, reducing T cell proliferation [68]. The phosphatase PGAM5 also plays a role here, where its dephosphorylation of the active site histidine of NME2 prevents NME2-driven activation of K_Ca_3.1, feeding into the inhibition of T-cell activation [69]. The pHis-dependent activity of K_Ca_3.1, regulated by (at least) two pHis phosphatases and a His kinase, neatly demonstrates a complex interplay of pHis signalling in mammalian cells.

In much the same way as some canonical phosphatases show dual specificity for pSer and pThr [70], the aforementioned histidine phosphatases PHPT1 and LHPP also display phosphatase activity towards pLys substrates. PHPT1 can dephosphorylate histone H1.2 chemically phosphorylated on Lys residues [71], while LHPP is active towards free 3-pHis and 6-pLys, as well as inorganic pyrophosphate [72], and even potentially Ser, Thr and Tyr [73,74]. Although LHPP appears to exhibit broad substrate specificity *in vitro* [75], to the best of our knowledge, *bona fide* LHPP substrates have yet to be identified. More generally, down-regulation of LHPP substantially increases pHis levels in cells and tissues [76] and it is known to play roles in tumour suppression [76,77], stem cell self-renewal [75] and depression [74]. It is worth noting that there is limited evidence of direct protein phosphatase activity for LHPP. The studies outlined here relied on knockout/in of LHPP, and it is therefore possible that the observed reduction in pHis was indirect. Hindupur and colleagues demonstrated direct dephosphorylation of pHis by LHPP on denatured cell lysate extracted proteins immobilised on a membrane [76], although this raises an alternate question as to the ability of this phosphatase to act on folded proteins [78]. Nevertheless, the last decade has seen a notable rise in the number of papers linking histidine phosphorylation with different cancers, with the vast majority citing the tumour-suppressive effects of LHPP. Indeed, in gastric cancer for example, high LHPP levels were associated with increased infiltration of antitumour immune cells, such as CD8^+^ T cells [79]. In contrast, reduced LHPP levels in e.g. breast cancer and hepatocellular carcinoma have been associated with reduced survival rates [76,80]. Consequently, LHPP is now considered a promising therapeutic target, and early-stage exploration has demonstrated that LHPP up-regulation with small activating RNAs reduces the proliferation and migration of hepatocellular carcinoma in a mouse model [81]. Collectively, these studies highlight the key, underexplored roles of pHis and particularly LHPP in cancer research.

While pHis/pLys phosphatases appear somewhat promiscuous in their choice of substrates, the single mammalian pArg phosphatase identified to date appears to be quite specific for pArg [82,83]. Isolated from rat liver, this 17 kDa enzyme is active towards free pArg but does not dephosphorylate pHis, pLys or thioester-linked phosphates [82], and has been shown to selectively dephosphorylate pArg-phosphorylated peptides within a mixture containing pThr and pTyr peptides [83]. However, it should be noted that alkaline phosphatase from bovine intestine is also able to dephosphorylate pArg peptides, albeit with reduced efficiency compared with a pTyr-containing peptide [83,84], although whether extracellular pArg sites exist for this secreted phosphatase remains unclear.

More generally, it remains possible that the dynamics of these phosphorylation events may not be directly regulated through enzymatic treatment, but by phosphate transfer to an acceptor residue, either driving signalling processes or acting as a ‘sink’ for removal. Indeed, it is well established in plants, prokaryotes and fungi that phosphate can transfer from His to Asp as part of the two-component signalling systems. It is therefore possible that such transfer events are prevalent in mammalian systems, or that acid-mediated hydrolysis in localised acidic environments may regulate these signalling events.

### Phosphorylation of acidic residues: pAsp and pGlu signalling

Along with pHis, pAsp forms the central phospho-relay system responsible for signal transduction in two-component systems [38]. However, due to their inherent instability [34] (Figure 1), defining the precise sites of modification and the biological functions of pAsp and pGlu, particularly in mammalian systems, has generally proven challenging for both high- and low-throughput analyses. That being said, there are instances of acyl phosphates in proteins, e.g. as active intermediates in adenosine triphosphatase and acetate kinase, that are stable under extreme acidic conditions [85].

Our early work defining sites of pAsp and pGlu on human proteins using low-resolution tandem MS suffered from high rates of mislocalisation (estimated at 61% and 69%, respectively), computed based on a decoy phosphoalanine search [5]. We believe these high mislocalisation rates were due partly to the data acquisition strategy, but also due to the lability of the phosphate group and the fact that search algorithms have not been developed to consider these PTMs, greatly impeding site assignment. Bearing this in mind, the proteins we identified were significantly enriched for nuclear/nucleolar/nucleoplasm proteins, specifically poly(A) RNA binding, with implications for roles of both pAsp and pGlu in RNA processing, export and/or stability, and for pGlu in chromatin regulation. A separate MS-based study investigating pHis and pAsp in human prostate cancer cells claimed to identify 80 pAsp proteins with diverse functions, including metabolism, protein folding and cell motility [86]. However, the authors did not try to mitigate factors in their workflow that are generally accepted to be detrimental to such LaPhs, namely heat and acid, and they failed to perform any type of phosphopeptide enrichment, which is highly unusual. Critically, data analysis was conducted with relatively large mass error tolerance windows (0.5 Da) and so these apparent examples of human pAsp proteins must be treated with some caution.

Outside of these potential roles for acyl phosphates, very little is known about the potential roles of pAsp, and particularly pGlu, in regulating mammalian protein function. pAsp is a recognised catalytic intermediate, notably in the haloacid dehalogenase (HAD) phosphatase/phosphomutase superfamily, where pAsp forms the active site nucleophile involved in substrate phosphate removal [87]. Within the HADs, P-type ATPases, which include the Ca^2+^-ATPase pump, Na^+^/K^+^-ATPase pump and H^+^/K^+^-ATPase pump [88], use pAsp as a molecular switch to drive ion gradients, as exemplified by the sarcoplasmic reticulum (SR) Ca^2+^-ATPase pump (extensively reviewed in [89]). Here, Ca^2+^ binding on the cytoplasmic side of the channel triggers a conformational change that allows Mg^2+^-ATP to access the ATP binding pocket, subsequently transferring its γ-phosphate onto Asp351. This action induces a rotational change in the channel structure, occluding the cytoplasmic side of the channel and releasing Ca^2+^ ions into the SR lumen. Subsequent water-induced hydrolysis of pAsp351, catalysed by nearby Glu183, returns the channel to the ground state, thereby closing the luminal side of the channel and resetting the sensor.

A related, but slightly different role for pAsp is demonstrated by the two atypical Ser/Thr kinases, Rio1 and Rio2. In both humans [90,91] and yeast [92,93], these enzymes play critical roles in ribosome biogenesis. Originally classified as kinases, Rio1 and Rio2 are now thought of as ATPases, working in a similar way to P-type ATPases through a pAsp-driven mechanism. Elegant structural studies by Ferreira-Cerca and colleagues [92,93] exploring the mechanisms of action of these two ATPases revealed that autophosphorylation of the conserved Asp is required for divalent metal-ion binding (resulting in ADP-Mg^2+^ and a pAsp intermediate in the active site), inducing a conformational change that permits interaction with the pre-40S ribosome [93]. ATPase activity then releases first Rio2 [93] then Rio1 [92], aiding correct maturation of the nascent 40S ribosome.

More directly, early biochemical experiments using purified rat NME1 demonstrated that it could transfer phosphate from its His auto-phosphorylation site to an Asp residue (Asp319) on aldolase C [94]. The authors postulated that pAsp319 could be involved in the tumour-suppressive effects of NME1 observed in cultured breast cancer MDA-MB-435 cells, given the correlation of the phosphate transfer activity with inhibition of cell motility [95], but this was not definitively ascertained. This potential role in cancer progression, however, coupled with the actions of Rio1/2 and P-type ATPases, effectively demonstrates a critical regulatory role of Asp phosphorylation in mammalian cells.

Alongside pLys and pArg, pGlu is one of the most elusive LaPh sites, with only two validated instances in the literature that we were able to identify. The first such report describes Glu phosphorylation in collagen α2 chains derived from chicken bone [85], but without any functional readout of its effects. The authors do note that survival of this theoretically highly labile site following acidic extraction and hydrolysis may suggest that the local environment of these pGlu sites could play important roles in their stability. In this particular instance, it is possible that the collagen triple helix potentially shields the pGlu site from hydrolysis. Whether this notion holds true for LaPhs in general will be interesting to investigate.

Perhaps more interesting is the observation of extensive phosphorylation on glutamate residues (≥8) within the N-terminus of the nuclear protein prothymosin α [96]. Here, it is thought to act as a high-energy store in the nucleus, specifically for the synthesis, processing or export of RNA [97,98]. Not only does the half-life of pGlu on prothymosin α decrease nearly 2-fold (from ~72 min) in quiescent cells, but it notably increases during S-phase [97,98]. Abrogation or inhibition of transcription, either due to M-phase arrest or with actinomycin D, stabilised pGlu such that the half-life increased nearly 2.5 fold [98]. The presence of pGlu on an abundant nuclear protein fits loosely with our own high-throughput proteomics data showing enrichment of pGlu proteins in the nucleus/nucleoplasm [5], where we in fact observed Glu phosphorylation of thymosin β4, within its N-terminus (pGlu11). The consequences of Glu phosphorylation here are unknown, but with thymosin β4 playing important roles in cytoskeletal (re-)organisation and cell migration [99,100], it will be extremely interesting to explore whether pGlu functions primarily as a means of energy storage and/or has more defined roles, such as in structural regulation.

### Phosphorylation of redox residues: pCys signalling

PTMs on Cys are plentiful, owing largely to the redox sensitivity and nucleophilicity of its side chain [101], and the occurrence of pCys is well documented as an enzyme intermediate for protein tyrosine phosphatases (PTP, reviewed in [102]). This enzyme superfamily is characterised by a C(X)_5_R active site motif, where nucleophilic attack from the active site Cys residue on the substrate pTyr residue generates the pCys intermediate. Unsurprisingly, mutation of the active site Cys completely inhibits PTP activity [103].

Within the PTP superfamily sits the PRL PTP family, which exemplifies different pCys functions. These phosphatases adhere to the active site motif of C(X)_5_R mentioned above, but unlike the other family members, have an Ala substitution in place of the Ser/Thr immediately adjacent to the catalytic Arg residue [104]. At this position, Ser/Thr normally promotes dephosphorylation of the pCys intermediate. However, the absence of this hydroxyl-containing residue in PRLs reduces hydrolysis of the catalytic pCys intermediate (substantially increasing the half-life), meaning that the PRL family members are in fact poor pTyr phosphatases. Although lacking known substrates [105], PRL proteins are often overexpressed in malignant tumours [106,107] and have been shown to regulate Mg^2+^ homeostasis through a pCys-dependent interaction with CNNM (CBS-pair domain divalent metal cation transport mediator) proteins [108]. In this scenario, CNNM4 promotes Mg^2+^ efflux from cells, but this is inhibited upon its interaction with PRL3. Phosphorylation of the active site Cys of PRL3, which is itself regulated in response to intracellular Mg^2+^ levels, interrupts this interaction, leaving CNNM4 free to stimulate Mg^2+^ efflux. Conversely, elevated Mg^2+^ levels increase PRL3 pCys, which disrupts the interaction with CNNM4, facilitating its ability to promote Mg^2+^ efflux [13,109]. Together, this evidence points to the PRL3-CNNM4 axis functioning as a sensor of free Mg^2+^ and regulator of Mg^2+^ homeostasis, which is likely responsible for the observed tumour-promoting activities of PRLs. However, the mechanism whereby the PRL active site Cys becomes phosphorylated in the first instance remains an unanswered question, given the lack of phosphatase activity and thus the availability of a phosphate donor.

Outside of the PTP protein family, reports of phosphorylated Cys are rare. We have previously identified over 50 unique sites of pCys in human proteins [5], including the mitochondrial kinase PTEN-induced kinase 1 (PINK1) (on Cys412), phosphorylation of which was subsequently validated in a separate study [110]. PINK1 is a mitochondrial kinase that accumulates on the surface of damaged mitochondria, marking them for mitophagy [111] in a process that requires PINK1-mediated phosphorylation of ubiquitin. Structural modelling of this pCys412 residue, which lies in the activation loop of PINK1, suggests that modification may inhibit ubiquitin binding of PINK1 [110]. Indeed, mutation of Cys412 to non-phosphorylatable Ala412 increased ubiquitin phosphorylation rates relative to wild-type enzyme, strongly suggesting a regulatory role for Cys412 phosphorylation on PINK1 activity, at least towards the substrate ubiquitin. Interestingly, Cys412 phosphorylation was also observed on kinase-inactive PINK1, suggesting this Cys phosphorylation is at least independent of PINK1’s own Ser/Thr kinase activity. Crucially, PINK1 is a key player in the pathology of early-onset Alzheimer’s disease [112], and thus a functional role for pCys here has the potential to open up new avenues of therapeutic intervention for neurodegenerative diseases.

Some of these studies, and others, mark out pCys as both acid- [14,113] and heat-labile [13], offering a potential explanation for the low reported instances of pCys phosphorylation. However, a handful of reports [15,114], along with our own unpublished observations, suggest that the stability of pCys may be greater than once thought, particularly at low pH. Using a standard, highly acidic phosphopeptide enrichment workflow, we demonstrated hypoxia-dependent phosphorylation of Cys on a key hypoxia response regulator, HIF-1α (hypoxia-inducible factor 1α) [15]. Location of this pCys site within the HIF-1α PAS-A (Per-Arnt-Sim-A) domain led us to hypothesise that pCys90 might play a role in HIF complex formation, thereby regulating the hypoxic response, although this has not yet been proven. One contributing factor to the low frequency of reported pCys modifications could be its relatively low frequency in eukaryotic protein sequences, with only the amino acid Trp being found less frequently than Cys [115]. Additionally, and as with the other mammalian LaPh sites, the vast majority of MS-based studies simply do not interrogate their data for phosphate-mediated modifications other than on Ser/Thr/Tyr. In the case of variable Cys modifications, this is likely compounded given the sample preparation and data searching workflows employed across the majority of high-throughput experiments: Cys residues are almost always alkylated to disrupt disulphide bonds and promote proteolytic efficiency. Consequently, there is a propensity to search these data considering alkylated Cys as a static modification, which would preclude the ability to identify other types of Cys modification, including pCys.

## Conclusions

**Figure 2:**
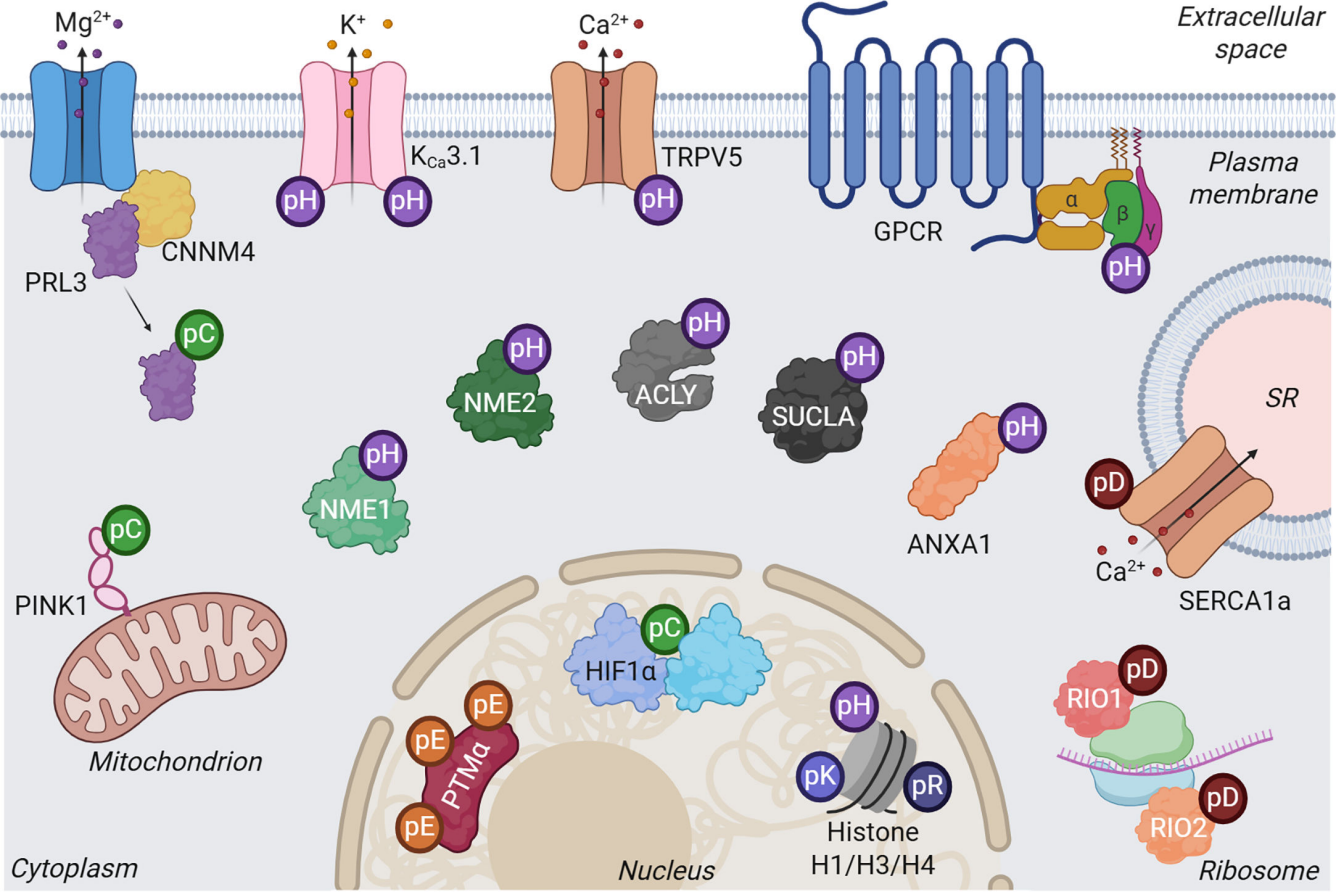
Cellular distribution of key known labile phosphosites. ACLY, ATP-citrate lyase; ANXA1, annexin A1; PTMα, prothymosin α; SERCA1a, sarcoplasmic reticulum Ca^2+^-ATPase 1 a; SR, sarcoplasmic reticulum; SUCLA, succinate-CoA ligase; pC, pCys; pD, pAsp; pE, pGlu; pH, pHis; pK, pLys; pR, pArg. Created in https://BioRender.com.

Examples of phosphorylation on all six LaPh residues (His, Lys, Arg, Asp, Glu and Cys) have been identified in various mammalian systems and discussed here (Figure 2). Reports of pHis modifications have undoubtedly been the most prevalent in recent years, arguably due to the development of tried and tested routes for making stable pHis mimetics [116,117], the production of pHis-specific antibodies, which have expanded the investigator’s toolbox [40,118], and the ability to readily generate pHis-containing peptides using a simple chemical reaction [45]. As we improve our ability to generate standards for analysis, incorporate LaPh residues into proteins artificially and generate PTM-specific antibodies, we will undoubtedly increase the pace of discovery. Nevertheless, a variety of roles have been either elucidated or proposed for LaPh sites, which raise intriguing questions as to their prevalence and function. While some are *bona fide* kinase substrates (best exemplified by His phosphorylation of NME1/2 targets), others exist as catalytic intermediates driving cellular processes and regulating signalling machinery (*cf.* pAsp and pCys phosphorylation, respectively). In the wider context, it may be that some phosphorylated residues, such as pGlu, act as energy stores rather than signalling molecules, or that some phosphosites arise due to phosphate transfer rather than active catalysis, as is the case with some sites of Cys phosphorylation. Irrespective, it will be important for the field to address this challenge head-on. If our focus remains solely on the classic phosphomonoesters, we are at risk of ignoring an untapped area of import for biological investigation, but perhaps more worryingly, incorrectly assigning site-specific functions to the classic phosphorylated residues through misidentification. Advances in sample preparation workflows, instrument sensitivity and data analysis pipelines should hopefully propel the field to new heights, and so uncover new regulatory mechanisms in health and disease that could generate opportunities for therapeutic intervention.

**Perspectives**

- Although generally downplayed by the phosphorylation signalling field, the variety of roles starting to be uncovered for LaPhs in mammalian cells has the potential to shed light on whole new areas of biological control. Potential regulation of mammalian signalling pathways opens up new avenues of druggable targets in disease areas such as cancer and neurodegeneration.
- Evidenced by high-throughput (mostly mass spectrometry-based) studies and traditional biochemical approaches, the current picture of mammalian LaPh functions in mammalian cells ranges from roles in epigenetics and DNA/RNA processing to energy and cation homeostasis.
- As our understanding of the physicochemical properties of LaPhs expands, and with it, the tools for their investigation, developments in sample preparation workflows, instrumentation and software for their characterisation, we anticipate that defining the key roles that LaPhs play in mammalian biology will become a mainstay in our understanding of phosphorylation-dependent signalling.

## Abbreviations

CNNM: CBS-pair domain divalent metal cation transport mediator
FAK: focal adhesion kinase
GPCR: G-protein couple receptor
HAD: haloacid dehalogenase
HIF1α: hypoxia-inducible factor 1α
KCa3.1: intermediate conductance calcium-activated potassium channel protein 4
LHPP: phospholysine phosphohistidine inorganic pyrophosphate phosphatase
LaPhs: labile phosphosites
MS: mass spectrometry
NDPK: nucleotide diphosphate kinase
NME: non-metastatic cells expressed in
PAS-A: Per-Arnt-Sim-A
PGAM5: phosphoglycerate mutase family member 5
PHPT1: phosphohistidine phosphatase 1
PINK1: PTEN-induced kinase 1
PRL: phosphatase of regenerating liver
PTM: post-translational modification
PTP: protein tyrosine phosphatase
SERCA1a: sarcoplasmic/endoplasmic reticulum Ca -ATPase 1a
SR: sarcoplasmic reticulum
TRPV5: transient receptor potential cation channel subfamily V member 5

## References

[1] Kalyuzhnyy A. Eyers P.A. Eyers C.E. Bowler-Barnett E. Martin M.J. Sun Z. et al 2022 Profiling the human phosphoproteome to estimate the true extent of protein phosphorylation J. Proteome Res. 21 1510 1524 10.1021/acs.jproteome.2c00131 35532924 PMC9171898

[2] Byford MF 1991 Rapid and selective modification of phosphoserine residues catalysed by Ba2+ ions for their detection during peptide microsequencing Biochem. J. 280 (Pt 1) 261 265 10.1042/bj2800261 1741751 PMC1130629

[3] Duclos B. Marcandier S. Cozzone AJ 1991 Chemical properties and separation of phosphoamino acids by thin-layer chromatography and/or electrophoresis Meth. Enzymol. 201 10 21 10.1016/0076-6879(91)01004-l 1943759

[4] Martensen T.M 1982 Phosphotyrosine in proteins Stability and quantification. J Biol Chem 257 9648 9652 10.1016/S0021-9258(18)34121-8 6179934

[5] Hardman G. Perkins S. Brownridge P.J. Clarke C.J. Byrne D.P. Campbell A.E. et al 2019 Strong anion exchange-mediated phosphoproteomics reveals extensive human non-canonical phosphorylation EMBO J. 38 e100847 10.15252/embj.2018100847 31433507 PMC6826212

[6] Kowalewska K. Stefanowicz P. Ruman T. Frączyk T. Rode W. Szewczuk Z 2010 Electron capture dissociation mass spectrometric analysis of lysine-phosphorylated peptides Biosci. Rep. 30 433 443 10.1042/BSR20090167 20144148 PMC2947194

[7] Huebner V.D. Matthews HR 1985 Phosphorylation of histidine in proteins by a nuclear extract of Physarum polycephalum plasmodia Journal of Biological Chemistry 260 16106 16113 10.1016/S0021-9258(17)36207-5 4066704

[8] Prust N. Van Breugel P.C. Lemeer S 2022 Widespread Arginine Phosphorylation in Staphylococcus aureus Mol. Cell Proteomics 21 100232 10.1016/j.mcpro.2022.100232 35421590 PMC9112008

[9] Schmidt A. Trentini D.B. Spiess S. Fuhrmann J. Ammerer G. Mechtler K. et al 2014 Quantitative phosphoproteomics reveals the role of protein arginine phosphorylation in the bacterial stress response Mol. Cell Proteomics 13 537 550 10.1074/mcp.M113.032292 24263382 PMC3916652

[10] Black S. Wright NG 1953 Enzymatic reduction of β-aspartyl phosphate to homoserine J. Am. Chem. Soc. 75 5766 5766 10.1021/ja01118a532

[11] Koshland DE 1952 Effect of catalysts on the hydrolysis of acetyl phosphate. nucleophilic displacement mechanisms in enzymatic reactions ^1^ . J. Am. Chem. Soc. 74 2286 2292 10.1021/ja01129a035

[12] Post R.L. Kume S 1973 Evidence for an aspartyl phosphate residue at the active site of sodium and potassium ion transport adenosine triphosphatase Journal of Biological Chemistry 248 6993 7000 10.1016/S0021-9258(19)43350-4 4270326

[13] Gulerez I. Funato Y. Wu H. Yang M. Kozlov G. Miki H. et al 2016 Phosphocysteine in the PRL-CNNM pathway mediates magnesium homeostasis EMBO Rep. 17 1890 1900 10.15252/embr.201643393 27856537 PMC5283600

[14] Meins M. Jenö P. Müller D. Richter W.J. Rosenbusch J.P. Erni B 1993 Cysteine phosphorylation of the glucose transporter of Escherichia coli Journal of Biological Chemistry 268 11604 11609 10.1016/S0021-9258(19)50244-7 8505292

[15] Daly L.A. Brownridge P.J. Batie M. Rocha S. Sée V. Eyers C.E 2021 Oxygen-dependent changes in binding partners and post-translational modifications regulate the abundance and activity of HIF-1α/2α Sci. Signal. 14 692 10.1126/scisignal.abf6685 34285132

[16] Morgan J.A.M. Singh A. Kurz L. Nadler-Holly M. Ruwolt M. Ganguli S. et al 2024 Extensive protein pyrophosphorylation revealed in human cell lines Nat. Chem. Biol. 20 1305 1316 10.1038/s41589-024-01613-5 38664588 PMC11427299

[17] Eyers C.E. Clarke CJ 2024 Pyro(phospho)mania Nat. Chem. Biol. 20 1248 1249 10.1038/s41589-024-01606-4 38664587

[18] Mihiret Y.E. Schaaf G. Kamleitner M 2024 Protein pyrophosphorylation by inositol phosphates: a novel post-translational modification in plants? Front. Plant Sci. 15 1347922 10.3389/fpls.2024.1347922 38455731 PMC10917965

[19] Penkert M. Yates L.M. Schümann M. Perlman D. Fiedler D. Krause E 2017 Unambiguous identification of serine and threonine pyrophosphorylation using neutral-loss-triggered electron-transfer/higher-energy collision dissociation Anal. Chem. 89 3672 3680 10.1021/acs.analchem.6b05095 28218834

[20] Azevedo C. Borghi F. Su X.B. Saiardi A 2024 On the covalent nature of lysine polyphosphorylation Mol. Cell 84 1811 1815 10.1016/j.molcel.2024.03.029 38701742

[21] Azevedo C. Livermore T. Saiardi A 2015 Protein polyphosphorylation of lysine residues by inorganic polyphosphate Mol. Cell 58 71 82 10.1016/j.molcel.2015.02.010 25773596

[22] Baijal K. Downey M 2021 The promises of lysine polyphosphorylation as a regulatory modification in mammals are tempered by conceptual and technical challenges Bioessays 43 e2100058 10.1002/bies.202100058 33998006

[23] Neville N. Lehotsky K. Klupt K.A. Downey M. Jia Z 2024 Polyphosphate attachment to lysine repeats is a non-covalent protein modification Mol. Cell 84 1802 1810 10.1016/j.molcel.2024.03.028 38701741

[24] Celik A. Schöpf F. Stieger C.E. Lampe S. Hanf B. Morgan J.A.M. et al 2025 Nucleoside diphosphate kinase A (NME1) catalyses its own oligophosphorylation Nat. Chem. 10.1038/s41557-025-01915-8 40835738 PMC12580328

[25] Neville N. Lehotsky K. Jia Z 2024 Back on the chain gang: polyphosphate modification of proteins Trends Biochem. Sci. 49 757 760 10.1016/j.tibs.2024.06.010 38945730

[26] Neville N. Lehotsky K. Yang Z. Klupt K.A. Denoncourt A. Downey M et al 2023 Modification of histidine repeat proteins by inorganic polyphosphate Cell Rep. 42 113082 10.1016/j.celrep.2023.113082 37660293

[27] Kühlberg A. Haid M. Metzger S 2010 Characterization of O-phosphohydroxyproline in rat {alpha}-crystallin A J. Biol. Chem. 285 31484 31490 10.1074/jbc.M109.035428 20682783 PMC2951222

[28] Feramisco J.R. Kemp B.E. Krebs E.G 1979 Phosphorylation of hydroxyproline in a synthetic peptide catalyzed by cyclic AMP-dependent protein kinase Journal of Biological Chemistry 254 6987 6990 10.1016/S0021-9258(18)50272-6 222752

[29] Urushizaki Y. Seifter S 1985 Phosphorylation of hydroxylysine residues in collagen synthesized by cultured aortic smooth muscle cells Proc. Natl. Acad. Sci. U.S.A. 82 3091 3095 10.1073/pnas.82.10.3091 3858806 PMC397720

[30] Piggott M.J. Attwood P.V 2017 Focus on O-phosphohydroxylysine, O-phosphohydroxyproline, N ^1^-phosphotryptophan and S-phosphocysteine Amino Acids 49 1309 1323 10.1007/s00726-017-2446-x 28578504

[31] Daly L.A. Clarke C.J. Po A. Oswald S.O. Eyers C.E 2023 Considerations for defining +80 Da mass shifts in mass spectrometry-based proteomics: phosphorylation and beyond Chem. Commun. (Camb.) 59 11484 11499 10.1039/d3cc02909c 37681662 PMC10521633

[32] Boyer P.D. DeLuca M. Ebner K.E. Hultquist D.E. Peter J.B 1962 Identification of phosphohistidine in digests from a probable intermediate of oxidative phosphorylation Journal of Biological Chemistry 237PC3306 PC3308 10.1016/S0021-9258(18)50167-8 14014715

[33] Besant P.G. Attwood P.V. Piggott M.J 2009 Focus on phosphoarginine and phospholysine Curr. Protein Pept. Sci. 10 536 550 10.2174/138920309789630598 19751195

[34] Attwood P.V. Besant P.G. Piggott MJ 2011 Focus on phosphoaspartate and phosphoglutamate Amino Acids 40 1035 1051 10.1007/s00726-010-0738-5 20859643

[35] Buchowiecka A.K 2014 Puzzling over protein cysteine phosphorylation--assessment of proteomic tools for S-phosphorylation profiling Analyst 139 4118 4123 10.1039/c4an00724g 25011562

[36] Ning J. Sala M. Reina J. Kalagiri R. Hunter T. McCullough B.S 2024 Histidine phosphorylation: protein kinases and phosphatases Int. J. Mol. Sci. 25 14 7975 10.3390/ijms25147975 39063217 PMC11277029

[37] Choi S. Lee S.H. Kee J.M 2025 Bringing Histidine Phosphorylation into Light: Role of Chemical Tools ACS Chem. Biol. 20 778 790 10.1021/acschembio.5c00103 40184269

[38] Alvarez A.F. Georgellis D 2022 The role of sensory kinase proteins in two-component signal transduction Biochem. Soc. Trans. 50 1859 1873 10.1042/BST20220848 36398786

[39] Cui F. Qian X. Ying W 2021 Integrated strategy for unbiased profiling of the histidine phosphoproteome Anal. Chem. 93 15584 15589 10.1021/acs.analchem.1c03374 34787389

[40] Fuhs S.R. Meisenhelder J. Aslanian A. Ma L. Zagorska A. Stankova M. et al 2015 Monoclonal 1- and 3-phosphohistidine antibodies: new tools to study histidine phosphorylation Cell 162 198 210 10.1016/j.cell.2015.05.046 26140597 PMC4491144

[41] Hu Y. Jiang B. Weng Y. Sui Z. Zhao B. Chen Y. et al 2020 Bis(zinc(II)-dipicolylamine)-functionalized sub-2 μm core-shell microspheres for the analysis of N-phosphoproteome Nat. Commun. 11 6226 10.1038/s41467-020-20026-1 33277485 PMC7718886

[42] Leijten N.M. Heck A.J.R. Lemeer S 2022 Histidine phosphorylation in human cells; a needle or phantom in the haystack? Nat. Methods 19 827 828 10.1038/s41592-022-01524-0 35726056

[43] Hunter T 2022 A journey from phosphotyrosine to phosphohistidine and beyond Mol. Cell 82 2190 2200 10.1016/j.molcel.2022.05.007 35654043 PMC9219344

[44] Makwana M.V. Williamson M.P. Jackson R.F.W. Muimo R 2022 Quantitation of phosphohistidine in proteins in a mammalian cell line by 31P NMR PLoS ONE 17 e0273797 10.1371/journal.pone.0273797 36048825 PMC9436146

[45] Wei Y.F. Matthews H.R 1991 Identification of phosphohistidine in proteins and purification of protein-histidine kinases Meth. Enzymol. 200 388 414 10.1016/0076-6879(91)00156-q 1956326

[46] Gonzalez-Sanchez M.B. Lanucara F. Hardman G.E. Eyers C.E 2014 Gas-phase intermolecular phosphate transfer within a phosphohistidine phosphopeptide dimer Int. J. Mass Spectrom. 367 28 34 10.1016/j.ijms.2014.04.015 25844054 PMC4375673

[47] Fuhs S.R. Hunter T 2017 pHisphorylation: the emergence of histidine phosphorylation as a reversible regulatory modification Curr. Opin. Cell Biol. 45 8 16 10.1016/j.ceb.2016.12.010 28129587 PMC5482761

[48] Freije J.M. Blay P. MacDonald N.J. Manrow R.E. Steeg P.S 1997 Site-directed mutation of Nm23-H1. Mutations lacking motility suppressive capacity upon transfection are deficient in histidine-dependent protein phosphotransferase pathways in vitro J. Biol. Chem. 272 5525 5532 10.1074/jbc.272.9.5525 9038158

[49] Wagner P.D. Vu ND 1995 Phosphorylation of ATP-citrate lyase by nucleoside diphosphate kinase J. Biol. Chem. 270 21758 21764 10.1074/jbc.270.37.21758 7665595

[50] Sheikh M.H. Solito E 2018 Annexin A1: uncovering the many talents of an old protein Int. J. Mol. Sci. 19 1045 10.3390/ijms19041045 29614751 PMC5979524

[51] Muimo R. Banner S.J. Marshall L.J. Mehta A 1998 Nucleoside diphosphate kinase and Cl(-)-sensitive protein phosphorylation in apical membranes from ovine airway epithelium Am. J. Respir. Cell Mol. Biol. 18 270 278 10.1165/ajrcmb.18.2.2850 9476915

[52] ——, Hornickova Z. Riemen C.E. Gerke V. Matthews H. Mehta A 2000 Histidine phosphorylation of annexin I in airway epithelia J. Biol. Chem. 275 36632 36636 10.1074/jbc.M000829200 10956639

[53] Zhang J. Gelman I.H. Qu J. Hochwald S.N 2023 Phosphohistidine signaling promotes FAK-RB1 interaction and growth factor-independent proliferation of esophageal squamous cell carcinoma Oncogene 42 449 460 10.1038/s41388-022-02568-4 36513743

[54] Cai X. Srivastava S. Surindran S. Li Z. Skolnik E.Y 2014 Regulation of the epithelial Ca Mol. Biol. Cell 25 1244 1250 10.1091/mbc.E13-04-0180 24523290 PMC3982990

[55] Srivastava S. Li Z. Ko K. Choudhury P. Albaqumi M. Johnson A.K. et al 2006 Histidine phosphorylation of the potassium channel KCa3.1 by nucleoside diphosphate kinase B is required for activation of KCa3.1 and CD4 T cells Mol. Cell 24 665 675 S1097-2765(06)00783-0 10.1016/j.molcel.2006.11.012 17157250

[56] Srivastava S. Panda S. Li Z. Fuhs S.R. Hunter T. Thiele D.J. et al 2016 Histidine phosphorylation relieves copper inhibition in the mammalian potassium channel KCa3.1 Elife 5 e16093 10.7554/eLife.16093 27542194 PMC5005030

[57] Cuello F. Schulze R.A. Heemeyer F. Meyer H.E. Lutz S. Jakobs K.H. et al 2003 Activation of heterotrimeric G proteins by a high energy phosphate transfer via nucleoside diphosphate kinase (NDPK) B and Gbeta subunits. Complex formation of NDPK B with Gbeta gamma dimers and phosphorylation of His-266 IN Gbeta J. Biol. Chem. 278 7220 7226 10.1074/jbc.M210304200 12486123

[58] Bertran-Vicente J. Serwa R.A. Schümann M. Schmieder P. Krause E. Hackenberger C.P.R 2014 Site-specifically phosphorylated lysine peptides J. Am. Chem. Soc. 136 13622 13628 10.1021/ja507886s 25196693

[59] Fu S. Fu C. Zhou Q. Lin R. Ouyang H. Wang M. et al 2020 Widespread arginine phosphorylation in human cells—a novel protein PTM revealed by mass spectrometry Sci. China Chem. 63 341 346 10.1007/s11426-019-9656-7

[60] Levy-Favatier F. Delpech M. Kruh J 1987 Characterization of an arginine-specific protein kinase tightly bound to rat liver DNA Eur. J. Biochem. 166 617 621 10.1111/j.1432-1033.1987.tb13558.x 3609029

[61] Wakim B.T. Aswad GD 1994 Ca(2+)-calmodulin-dependent phosphorylation of arginine in histone 3 by a nuclear kinase from mouse leukemia cells J. Biol. Chem. 269 2722 2727 8300603

[62] Besant P.G. Attwood PV 2012 Histone H4 histidine phosphorylation: kinases, phosphatases, liver regeneration and cancer Biochem. Soc. Trans. 40 290 293 10.1042/BST20110605 22260708

[63] Chen C.C. Bruegger B.B. Kern C.W. Lin Y.C. Halpern R.M. Smith RA 1977 Phosphorylation of nuclear proteins in rat regenerating liver Biochemistry 16 4852 4855 10.1021/bi00641a016 20941

[64] Chen C.C. Smith D.L. Bruegger B.B. Halpern R.M. Smith RA 1974 Occurrence and distribution of acid-labile histone phosphates in regenerating rat liver Biochemistry 13 3785 3789 10.1021/bi00715a026 4853420

[65] Klumpp S. Bechmann G. Mäurer A. Selke D. Krieglstein J 2003 ATP-citrate lyase as a substrate of protein histidine phosphatase in vertebrates Biochem. Biophys. Res. Commun. 306 110 115 10.1016/S0006-291X(03)00920-3 12788074

[66] Beckman-Sundh U. Ek B. Zetterqvist O. Ek P 2011 A screening method for phosphohistidine phosphatase 1 activity Ups. J. Med. Sci. 116 161 168 10.3109/03009734.2011.585253 21679093 PMC3128721

[67] Mäurer A. Wieland T. Meissl F. Niroomand F. Mehringer R. Krieglstein J. et al 2005 The β-subunit of G proteins is a substrate of protein histidine phosphatase Biochem. Biophys. Res. Commun. 334 1115 1120 10.1016/j.bbrc.2005.06.200 16039992

[68] Srivastava S. Zhdanova O. Di L. Li Z. Albaqumi M. Wulff H. et al 2008 Protein histidine phosphatase 1 negatively regulates CD4 T cells by inhibiting the K ^+^ channel KCa3.1 . Proc. Natl. Acad. Sci. U.S.A 105 14442 14446 10.1073/pnas.0803678105 18796614 PMC2538450

[69] Panda S. Srivastava S. Li Z. Vaeth M. Fuhs S.R. Hunter T. et al 2016 Identification of PGAM5 as a mammalian protein histidine phosphatase that plays a central role to negatively regulate CD4 + T Cells Mol. Cell 63 457 469 10.1016/j.molcel.2016.06.021 27453048 PMC5677525

[70] Brautigan D.L. Shenolikar S 2018 Protein Serine/Threonine Phosphatases: Keys to Unlocking Regulators and Substrates Annu. Rev. Biochem. 87 921 964 10.1146/annurev-biochem-062917-012332 29925267

[71] Ek P. Ek B. Zetterqvist Ö 2015 Phosphohistidine phosphatase 1 (PHPT1) also dephosphorylates phospholysine of chemically phosphorylated histone H1 and polylysine Ups. J. Med. Sci. 120 20 27 10.3109/03009734.2014.996720 25574816 PMC4389004

[72] Hiraishi H. Yokoi F. Kumon A 1998 3-phosphohistidine and 6-phospholysine are substrates of a 56-kDa inorganic pyrophosphatase from bovine liver Arch. Biochem. Biophys. 349 381 387 10.1006/abbi.1997.0480 9448729

[73] Chen M.J. Dixon J.E. Manning G 2017 Genomics and evolution of protein phosphatases Sci. Signal. 10 474 eaag1796 10.1126/scisignal.aag1796 28400531

[74] Zhuang L. Gao W. Chen Y. Fang W. Lo H. Dai X. et al 2024 LHPP in glutamatergic neurons of the ventral hippocampus mediates depression-like behavior by dephosphorylating CaMKIIα and ERK Biol. Psychiatry Cogn. Neurosci. Neuroimaging 95 389 402 10.1016/j.biopsych.2023.08.026 37678540

[75] Xia R.M. Yao D.B. Cai X.M. Xu X.Q 2021 LHPP-mediated histidine dephosphorylation suppresses the self-renewal of mouse embryonic stem cells Front. Cell Dev. Biol. 9 638815 10.3389/fcell.2021.638815 33796530 PMC8007871

[76] Hindupur S.K. Colombi M. Fuhs S.R. Matter M.S. Guri Y. Adam K. et al 2018 The protein histidine phosphatase LHPP is a tumour suppressor Nature 555 678 682 10.1038/nature26140 29562234 PMC6376988

[77] Wu F. Ma H. Wang X. Wei H. Zhang W. Zhang Y 2022 The histidine phosphatase LHPP: an emerging player in cancer Cell Cycle 21 1140 1152 10.1080/15384101.2022.2044148 35239447 PMC9103355

[78] Gohla A 2019 Do metabolic HAD phosphatases moonlight as protein phosphatases? Biochim. Biophys. Acta Mol. Cell Res. 1866 153 166 10.1016/j.bbamcr.2018.07.007 30030002

[79] Gao Y.-X. Guo X.-J. Lin B. Huang X.-B. Tu R.-H. Lin M. et al 2025 Targeting LHPP in neoadjuvant chemotherapy resistance of gastric cancer: insights from single-cell and multi-omics data on tumor immune microenvironment and stemness characteristics Cell Death Dis. 16 306 10.1038/s41419-025-07614-z 40240758 PMC12003742

[80] Liu X. Yu D. Yu Z. Su S. Jiang M. Zhao C 2025 LHPP-P38 MAPK/ERK-ETS1 Axis negative feedback signaling restrains progression in breast cancer Cancer Sci. 116 923 935 10.1111/cas.16448 39789996 PMC11967269

[81] Bi C.Q. Kang T. Qian Y.K. Kang M. Zeng X.H. Li LC 2024 Upregulation of LHPP by saRNA inhibited hepatocellular cancer cell proliferation and xenograft tumor growth PLoS ONE 19 e0299522 10.1371/journal.pone.0299522 38696452 PMC11065268

[82] Kuba M. Ohmori H. Kumon A 1992 Characterization of N omega-phosphoarginine hydrolase from rat liver Eur. J. Biochem. 208 747 752 10.1111/j.1432-1033.1992.tb17243.x 1327768

[83] Kumon A. Kodama H. Kondo M. Yokoi F. Hiraishi H 1996 N(omega)-phosphoarginine phosphatase (17 kDa) and alkaline phosphatase as protein arginine phosphatases J. Biochem. 119 719 724 10.1093/oxfordjournals.jbchem.a021301 8743574

[84] Nishino M. Tsujimura S. Kuba M. Kumon A 1994 N omega-phosphoarginine phosphatase from rat renal microsome was alkaline phosphatase Arch. Biochem. Biophys. 312 101 106 10.1006/abbi.1994.1286 8031115

[85] Cohen-Solal L. Cohen-Solal M. Glimcher MJ 1979 Identification of gamma-glutamyl phosphate in the alpha 2 chains of chicken bone collagen Proc. Natl. Acad. Sci. U.S.A. 76 4327 4330 10.1073/pnas.76.9.4327 291967 PMC411567

[86] Lapek J.D. Jr Tombline G. Kellersberger K.A. Friedman M.R. Friedman AE 2015 Evidence of histidine and aspartic acid phosphorylation in human prostate cancer cells Naunyn Schmiedebergs Arch. Pharmacol. 388 161 173 10.1007/s00210-014-1063-4 25373728

[87] Seifried A. Schultz J. Gohla A 2013 Human HAD phosphatases: structure, mechanism, and roles in health and disease FEBS J. 280 549 571 10.1111/j.1742-4658.2012.08633.x 22607316

[88] Kühlbrandt W 2004 Biology, structure and mechanism of P-type ATPases Nat. Rev. Mol. Cell Biol. 5 282 295 10.1038/nrm1354 15071553

[89] Toyoshima C 2009 How Ca2+-ATPase pumps ions across the sarcoplasmic reticulum membrane Biochim. Biophys. Acta 1793 941 946 10.1016/j.bbamcr.2008.10.008 19010358

[90] Widmann B. Wandrey F. Badertscher L. Wyler E. Pfannstiel J. Zemp I. et al 2012 The kinase activity of human Rio1 is required for final steps of cytoplasmic maturation of 40S subunits Mol. Biol. Cell 23 22 35 10.1091/mbc.E11-07-0639 22072790 PMC3248900

[91] Zemp I. Wild T. O’Donohue M.-F. Wandrey F. Widmann B. Gleizes P.-E. et al 2009 Distinct cytoplasmic maturation steps of 40S ribosomal subunit precursors require hRio2 J. Cell Biol. 185 1167 1180 10.1083/jcb.200904048 19564402 PMC2712965

[92] Ferreira-Cerca S. Kiburu I. Thomson E. LaRonde N. Hurt E 2014 Dominant Rio1 kinase/ATPase catalytic mutant induces trapping of late pre-40S biogenesis factors in 80S-like ribosomes Nucleic Acids Res. 42 8635 8647 10.1093/nar/gku542 24948609 PMC4117770

[93] Ferreira-Cerca S. Sagar V. Schäfer T. Diop M. Wesseling A.-M. Lu H. et al 2012 ATPase-dependent role of the atypical kinase Rio2 on the evolving pre-40S ribosomal subunit Nat. Struct. Mol. Biol. 19 1316 1323 10.1038/nsmb.2403 23104056 PMC3515705

[94] Wagner P.D. Vu ND 2000 Histidine to aspartate phosphotransferase activity of nm23 proteins: phosphorylation of aldolase C on Asp-319 Biochem. J. 346 623 630 10.1042/bj3460623 10698688 PMC1220894

[95] MacDonald N.J. Freije J.M. Stracke M.L. Manrow R.E. Steeg P.S 1996 Site-directed mutagenesis of nm23-H1. Mutation of proline 96 or serine 120 abrogates its motility inhibitory activity upon transfection into human breast carcinoma cells J. Biol. Chem. 271 25107 25116 10.1074/jbc.271.41.25107 8810265

[96] Trumbore M.W. Wang R.H. Enkemann S.A. Berger SL 1997 Prothymosin α in vivo contains phosphorylated glutamic acid residues Journal of Biological Chemistry 272 26394 26404 10.1074/jbc.272.42.26394 9334214

[97] Wang R.H. Tao L. Trumbore M.W. Berger SL 1997 turnover of the acyl phosphates of human and murine prothymosin α in Vivo Journal of Biological Chemistry 272 26405 26412 10.1074/jbc.272.42.26405 9334215

[98] Tao L. Wang R.H. Enkemann S.A. Trumbore M.W. Berger SL 1999 Metabolic regulation of protein-bound glutamyl phosphates: insights into the function of prothymosin alpha J. Cell. Physiol. 178 154 163 10.1002/(SICI)1097-4652(199902)178:2<154::AID-JCP4>3.0.CO;2-V 10048579

[99] Scheller I. Beck S. Göb V. Gross C. Neagoe R.A.I. Aurbach K. et al 2022 Thymosin β4 is essential for thrombus formation by controlling the G-actin/F-actin equilibrium in platelets Haematologica 107 2846 2858 10.3324/haematol.2021.278537 34348450 PMC9713564

[100] Ying Y. Tao N. Zhang F. Wen X. Zhou M. Gao J 2024 Thymosin β4 regulates the differentiation of thymocytes by controlling the cytoskeletal rearrangement and mitochondrial transfer of thymus epithelial cells Int. J. Mol. Sci. 25 1088 10.3390/ijms25021088 38256161 PMC10816181

[101] Boutin C. Clément C. Rivoal J 2024 Post-Translational Modifications to Cysteine Residues in Plant Proteins and Their Impact on the Regulation of Metabolism and Signal Transduction Int. J. Mol. Sci. 25 18 9845 10.3390/ijms25189845 39337338 PMC11432348

[102] Tautz L. Critton D.A. Grotegut S 2013 Protein tyrosine phosphatases: structure, function, and implication in human disease Methods Mol. Biol. 1053 179 221 10.1007/978-1-62703-562-0_13 23860656 PMC8158066

[103] Zhang ZY 2002 Protein tyrosine phosphatases: structure and function, substrate specificity, and inhibitor development Annu. Rev. Pharmacol. Toxicol. 42 209 234 10.1146/annurev.pharmtox.42.083001.144616 11807171

[104] Kozlov G. Cheng J. Ziomek E. Banville D. Gehring K. Ekiel I 2004 Structural insights into molecular function of the metastasis-associated phosphatase PRL-3 J. Biol. Chem. 279 11882 11889 10.1074/jbc.M312905200 14704153

[105] Rios P. Li X. Köhn M 2013 Molecular mechanisms of the PRL phosphatases FEBS J. 280 505 524 10.1111/j.1742-4658.2012.08565.x 22413991

[106] Bessette D.C. Qiu D. Pallen C.J 2008 PRL PTPs: mediators and markers of cancer progression Cancer Metastasis Rev. 27 231 252 10.1007/s10555-008-9121-3 18224294

[107] Saha S. Bardelli A. Buckhaults P. Velculescu V.E. Rago C. St Croix B. et al 2001 A phosphatase associated with metastasis of colorectal cancer Science 294 1343 1346 10.1126/science.1065817 11598267

[108] Yoshida A. Funato Y. Miki H 2018 Phosphatase of regenerating liver maintains cellular magnesium homeostasis Biochem. J. 475 1129 1139 10.1042/BCJ20170756 29487165

[109] Zhang H. Kozlov G. Li X. Wu H. Gulerez I. Gehring K 2017 PRL3 phosphatase active site is required for binding the putative magnesium transporter CNNM3 Sci. Rep. 7 48 10.1038/s41598-017-00147-2 28246390 PMC5427921

[110] Waddell A.D. Ojha H. Agarwal S. Clarke C.J. Terriente-Felix A. Zhou H et al 2023 Regulation of Human PINK1 ubiquitin kinase by Serine167, Serine228 and Cysteine412 phosphorylation bioRxiv 10.1101/2023.03.31.534916

[111] Goodall E.A. Kraus F. Harper J.W 2022 Mechanisms underlying ubiquitin-driven selective mitochondrial and bacterial autophagy Mol. Cell 82 1501 1513 10.1016/j.molcel.2022.03.012 35364016 PMC9254164

[112] Valente E.M. Abou-Sleiman P.M. Caputo V. Muqit M.M.K. Harvey K. Gispert S. et al 2004 Hereditary early-onset Parkinson’s disease caused by mutations in PINK1 Science 304 1158 1160 10.1126/science.1096284 15087508

[113] Pas H.H. Robillard GT 1988 S-Phosphocysteine and phosphohistidine are intermediates in the phosphoenolpyruvate-dependent mannitol transport catalyzed by Escherichia coli EIImtl Biochemistry 27 5835 5839 10.1021/bi00416a002 3142516

[114] Sun F. Ding Y. Ji Q. Liang Z. Deng X. Wong C.C.L. et al 2012 Protein cysteine phosphorylation of SarA/MgrA family transcriptional regulators mediates bacterial virulence and antibiotic resistance Proc. Natl. Acad. Sci. U.S.A 109 15461 15466 10.1073/pnas.1205952109 22927394 PMC3458358

[115] Pe’er I. Felder C.E. Man O. Silman I. Sussman J.L. Beckmann JS 2004 Proteomic signatures: amino acid and oligopeptide compositions differentiate among phyla Proteins 54 20 40 10.1002/prot.10559 14705021

[116] Adam K. Hunter T 2018 Histidine kinases and the missing phosphoproteome from prokaryotes to eukaryotes Lab. Invest. 98 233 247 10.1038/labinvest.2017.118 29058706 PMC5815933

[117] Kee J.M. Villani B. Carpenter L.R. Muir TW 2010 Development of stable phosphohistidine analogues J. Am. Chem. Soc. 132 14327 14329 10.1021/ja104393t 20879710 PMC3189792

[118] Kalagiri R. Stanfield R.L. Meisenhelder J. La Clair J.J. Fuhs S.R. Wilson I.A. et al 2021 Structural basis for differential recognition of phosphohistidine-containing peptides by 1-pHis and 3-pHis monoclonal antibodies Proc. Natl. Acad. Sci. U.S.A. 118 e2010644118 10.1073/pnas.2010644118 33547238 PMC8017925

